# Hyperkinetic Choreiform Movements Secondary to Basal Ganglia Calcification and Underlying Developmental Venous Anomaly

**DOI:** 10.7759/cureus.22752

**Published:** 2022-03-01

**Authors:** Jay Patel, Muhammad Khalil, Sidra Zafar

**Affiliations:** 1 Internal Medicine, Orange Park Medical Center, Orange Park, USA

**Keywords:** calcifications, basal ganglia, deep venous anomaly, movement disorders, chorea

## Abstract

Chorea is irregular, involuntary movements that are associated with a variety of conditions and can encompass a wide differential. Unilateral hyperkinetic choreiform movements represent a rare subset characterized by the acute and sudden onset of progressive, uncontrollable body movements. We present a patient presenting with left-sided arm and leg arrhythmic movements caused by right-sided basal ganglia calcifications associated with an underlying developmental venous anomaly (DVA). To the author’s knowledge, this is only the second reported case of such a presentation, and it should be on the differential in patients presenting with unilateral movement abnormalities.

## Introduction

Movement disorders are present with a variety of manifestations and encompass a wide differential. Chorea is a subset of movement disorders, typically classified as random, fast, involuntary, and irregular movements with a writhing quality. Etiologies include hereditary causes such as Huntington’s disease. Nonhereditary causes include Sydenham, autoimmune, drug-induced, toxic, and metabolic such as hyperglycemia. Vascular causes have been presented, and chorea has been seen as one of the most common movement disorders presenting after strokes, particularly when the subthalamic nucleus is involved [1]. Unilateral chorea, in particular, has been associated with vascular causes, such as arteriovenous malformations and subdural hematomas, in addition to strokes [2,3]. 

The basal ganglia play a crucial role in the development of hyperkinetic movements through an imbalance of direct and indirect pathways [1]. Unilateral hyperkinetic movements associated with underlying pathology of the basal ganglia are primarily reported in conjunction with prior strokes [3]. Acute onset without any of the underlying risk factors mentioned above has not been thoroughly established.

Developmental venous anomalies are benign, congenital malformations of veins in the brain. Often discovered incidentally on imaging, they drain the normal parenchyma of the brain with a converging, abnormal system of veins that come together into a large trunk. In a retrospective analysis of 31 patients with developmental venous anomaly (DVA), computerized tomography (CT) angiography and magnetic resonance imaging (MRI) with contrast yielded detailed findings of the DVA, with CT without contrast and MR angiography deemed unable to contribute to identification. On MRI, the collector's vein is observed as a linear, small structure on all sequences. Contrast enhances the DVA with an enhancement of the venous collector and medullary vein due to slower flow [4].

Although common, DVA’s are typically benign and asymptomatic. However, with advancing age they can progress, with ischemic and hemorrhagic stroke being rare complications [5,6]. Cerebral calcifications in association with DVA are infrequent in the literature. Prior case reports primarily associate them with strokes, with only one prior report of an associated movement disorder. It has been hypothesized that hypertension in the venous network in the areas drained by DVA’s can contribute to encephalomalacia and gliosis, leading to eventual calcification [3]. 

We present an interesting case of a patient who presented with unilateral hyperkinetic chorea-like movements. They were found to have contralateral basal ganglia calcifications with an underlying DVA. Symptomatic unilateral chorea has prior only been reported in the setting of acute stroke. Although basal ganglia calcifications associated with DVA have been reported in the literature, there is only one other report of a concurrent chorea-like movement disorder [3].

## Case presentation

Our patient was a 58-year-old Caucasian female with a past medical history of coronary artery disease, hypertension, and type 2 diabetes mellitus who presented as a transfer from a rural hospital with chief complaints of uncontrollable, left-sided body movements and headache. Per the patient, the symptoms began one month prior to presentation with a diffuse headache and uncontrollable movements of her left foot. The patient was primarily thought to have subsequently and managed for restless leg syndrome by her primary care physician, suspecting to be the reason for her late presentation. Over time, the movements progressed up her entire left leg, and similar symptoms subsequently appeared in her left upper extremity. She described the movements as “random, uncontrollable, and jerk-like in nature.” The left side of her body had become fatigued and sore due to the constant motion. She denied focal weakness, facial droop, visual changes, sensory changes, dizziness, fall/trauma, or loss of consciousness. She denied any family history of neurological disease or a personal history of drug use. The symptoms had been causing the patient substantial physical and mental distress.

The patient would initially be sent to a local primary hospital by her primary care provider for further evaluation. Per the documentation, initial CT of the brain indicated no evidence of acute hemorrhage/stroke with abnormal calcifications noted in the basal ganglia region, with underlying neoplasm unable to be excluded and recommendations for further clarification with MRI. The patient was transferred to our hospital for further management and evaluation.

On presentation to our hospital, the patient was noted to be in no acute distress with stable vital signs. On exam, the patient was alert and oriented with appropriate speech and no obvious visual deficits. The patient had no active motor or sensory deficits in her face or bilateral extremities. The physical exam was primarily notable for the unilateral random, arrhythmic, uncontrollable, jerk-like movements of her left arm and leg. Movements were occurring both at rest and with active movement. Movements were random, with no inciting or alleviating factors, and they did not subside with distraction. Out of initial concern for possible underlying brain mass, neurosurgery was consulted, who recommended starting dexamethasone 4 mg IV every four hours and following up on the MRI.

**Figure 1:**
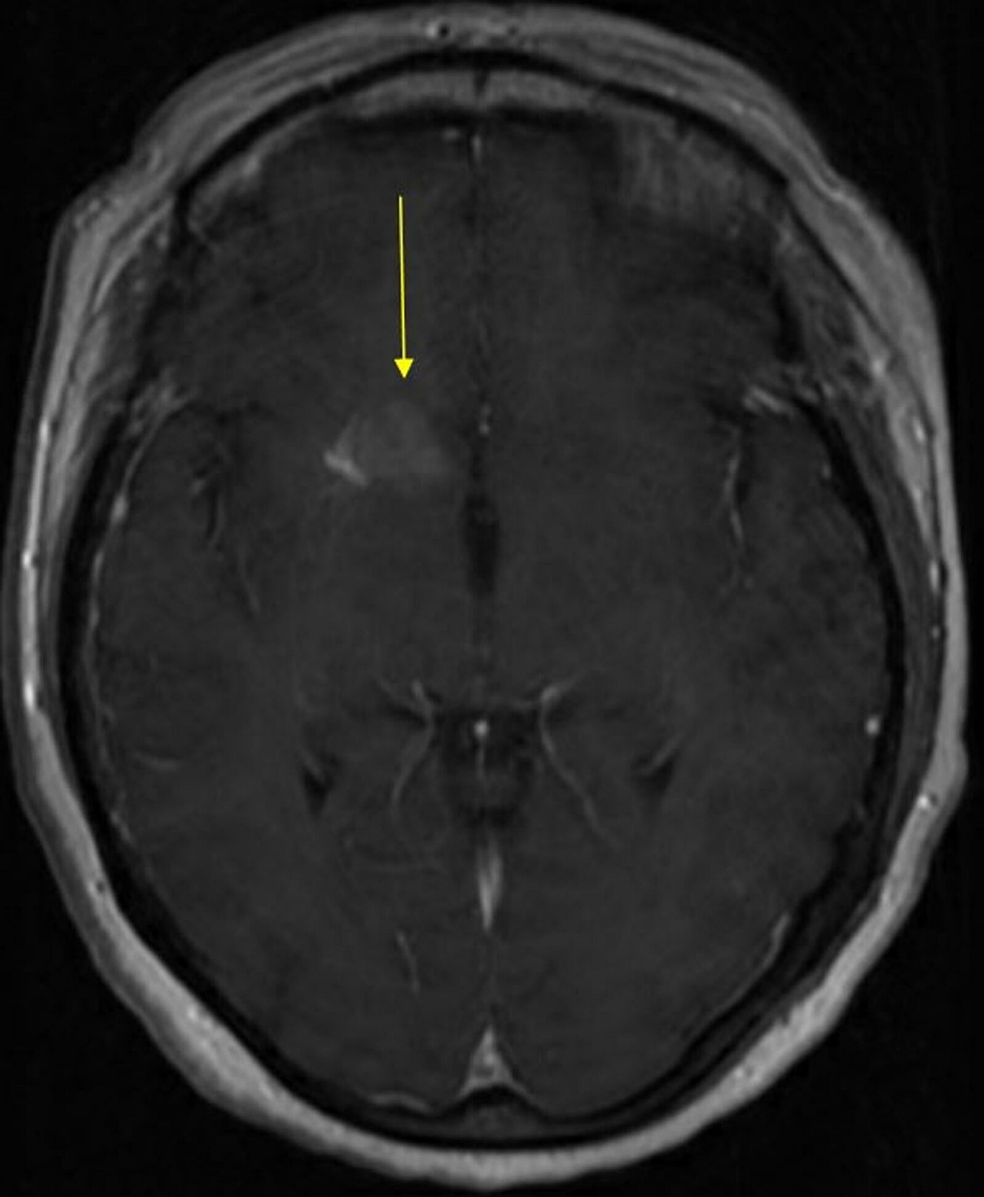
MRI brain with and without contrast, depicting unilateral dense calcification in the right basal ganglia, most likely corresponding to a deep venous anomaly (arrow). MRI: magnetic resonance imaging.

**Figure 2:**
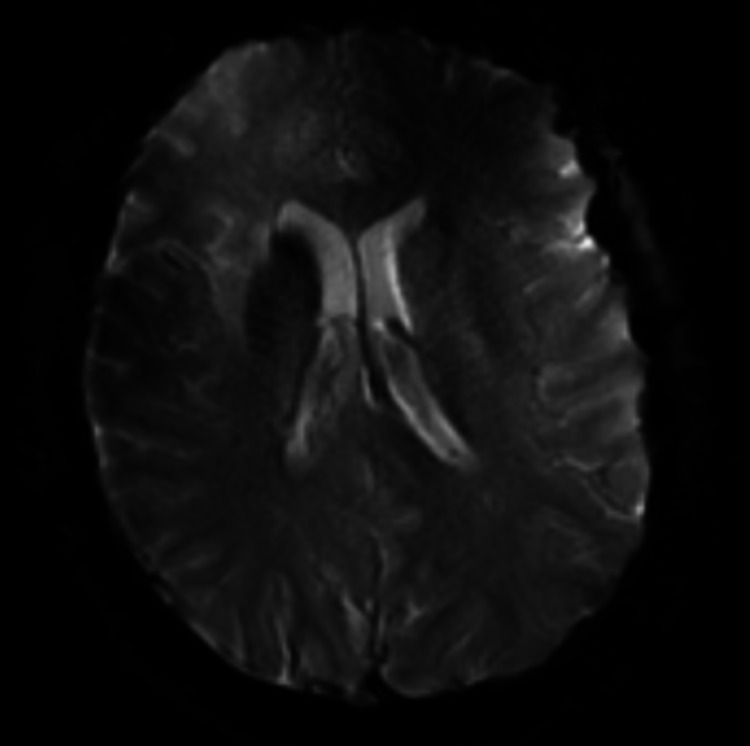
MRI gradient echo demonstrating hypointensity within the right basal ganglia reflecting calcification. MRI: magnetic resonance imaging.

Labs were noted to have a normal metabolic panel, with normal calcium, parathyroid hormone, and thyroid function. Hemoglobin A1C was 8%, with blood glucose on admission being 110 mg/dl. In addition, thyroid-stimulating hormone (TSH), anti-nuclear antibody (ANA), rapid plasma regain (RPR), and urine drug screen testing were negative. Urinalysis did not have evidence of infection nor proteinuria. Brain magnetic resonance imaging would be notable for right-sided unilateral calcifications within the basal ganglia, corresponding to a likely developmental venous anomaly, and nonspecific white matter changes. No anterior limb of the internal capsule involvement was noted. There was no evidence of acute or chronic stroke (Figures 1, 2).

Although initial concerns were for potential acute cerebrovascular accident or mass lesion, subsequent MRI did not have evidence of stroke nor mass lesion. Considering his left-sided deficits were lateralized contralateral to the findings of right-sided basal ganglia calcifications, it was hypothesized that his symptoms could therefore be related. After initially failing a baclofen trial, the patient responded with severe mitigation of symptoms to an initial dose of clonazepam 0.25 mg twice daily, which was increased to 0.5 mg twice daily on discharge with near-resolution of her symptoms. The patient was scheduled to follow-up with her primary care physician and a neurologist at discharge for ongoing management. At follow-up a few weeks after discharge, the patient was still being maintained on clonazepam therapy with resolution of her symptoms.

## Discussion

DVA's and basal ganglia calcifications have been prior reported in the literature. Concurrent movement disorders have primarily been associated in the setting of a recent stroke. Chorea-like hyperkinetic movements in association with these imaging findings have not been thoroughly reported or established.

Our patient had no evidence of stroke nor mass lesion on imaging, no family history suggestive of a hereditary cause, nor any obvious metabolic, drug-induced, or toxic insults. A common cause of chorea-like Huntington’s disease seemed unlikely considering the lack of family history, unilateral symptoms, and no executive or psychiatric deficits. The patient was not noted to be persistently or significantly hyperglycemic. TSH, ANA, and RPR testing were negative. Overall, autoimmune and vasculitis etiologies seemed unlikely in the absence of other systemic findings. There was no skin, joint, or kidney involvement noted. Outside of stroke, unilateral symptoms would not be expected in any other etiology [1].

Imaging indicated the presence of right-sided unilateral calcifications within the basal ganglia with associated DVA, which correlated with the contralateral presenting left-sided symptoms. As presented above, hyperkinetic movements in these circumstances typically occur in the setting of acute stroke, which was not evident in our patient. CT and subsequent MRI would have no evidence of acute or chronic stroke.

We found only one other reported case of similar movements associated with cerebral calcifications and DVA [3]. After a review of the success of clonazepam therapy in that reported case and associated literature, it was deemed appropriate to also initiate a trial in our patient, with almost near-resolution of symptoms observed.

Unilateral choreiform movements are rarely reported outside of stroke-caused injury to the basal ganglia [1]. The basal ganglia are a crucial component of the thalamic-inhibitory pathway. Any insult could theoretically result in a reduction in gamma-aminobutyric acid (GABA) transport to the globus pallidus, which would lead to over-activation of the system with the end result of possible hyperkinetic movements [7].

To our knowledge, this is the second reported case of basal ganglia calcifications with DVA associated with unilateral choreiform movements. We report a rare presentation that should be kept on the differential in patients who present with unilateral chorea. It is likely a diagnosis of exclusion, with emphasis on ruling out structural, genetic, auto-immune, and vasculitis causes, among others. Our patient responded well with clonazepam therapy, which should be considered for similar patients.

## Conclusions

Unilateral choreiform movements in association with basal ganglia calcification and DVA represent a rare presentation in patients who present with movement disorders. After ruling out hereditary, medication-induced, toxic, metabolic, and other known causes, brain imaging should be used in conjunction with history to deduce causes. Benzodiazepine therapy can be considered in these patients for symptom mitigation.
